# Does Intergenerational Social Mobility Affect Health and Mortality Risks Among Older Adults?

**DOI:** 10.1093/geroni/igaf122.2454

**Published:** 2025-12-31

**Authors:** Yuning Xie, Chen Bai

**Affiliations:** Renmin University of China, Beijing, United States; Renmin University of China, Beijing, Beijing, China (People’s Republic)

## Abstract

The health effects of intergenerational social mobility—particularly their existence, direction, and magnitude—remain subjects of ongoing debate, especially among older adults. This study leverages data from the 2017–2018 Chinese Longitudinal Healthy Longevity Survey – Healthy Family (CLHLS-HF) to investigate the impact of upward intergenerational occupational and educational mobility on health reproduction and to examine whether such mobility is significantly associated with mortality risk over a three-year period. The findings reveal that intergenerational social mobility exerts significant health effects, with upward occupational or educational mobility enhancing multidimensional health outcomes. Using a Cox proportional hazards model, the study finds that elderly individuals whose parents were farmers (or unemployed) and those who experienced greater absolute mobility distances exhibited significantly lower mortality risk. Additionally, a reversal in the number of chronic diseases was observed among elderly individuals whose final occupational status was “cadre,” along with a protective health effect associated with short-distance downward intergenerational occupational mobility. Robustness checks and instrumental variable (IV) tests confirm the reliability of these results. Furthermore, heterogeneity analysis indicates that the health benefits of intergenerational social mobility are more pronounced among younger elderly individuals. Overall, while the long-term health benefits of upward intergenerational social mobility are evident, certain “mixed” effects persist, warranting further investigation.

